# A nomogram for reduced cardiac function in postoperative acute type A aortic dissection patients with acute kidney injury undergoing continuous renal replacement therapy

**DOI:** 10.3389/fcvm.2022.874715

**Published:** 2022-07-22

**Authors:** Rui Jiao, Maomao Liu, Xuran Lu, Junming Zhu, Lizhong Sun, Nan Liu

**Affiliations:** ^1^Beijing Institute of Heart, Lung and Blood Vessel Diseases, Beijing Anzhen Hospital, Capital Medical University, Beijing, China; ^2^Center for Cardiac Intensive Care, Beijing Anzhen Hospital, Capital Medical University, Beijing, China; ^3^Beijing Aortic Disease Center, Beijing Anzhen Hospital, Capital Medical University, Beijing, China

**Keywords:** acute kidney injury, acute type A aortic dissection, continuous renal replacement therapy, postoperative ejection fraction reduction, diagnostic model

## Abstract

**Background:**

This study aimed to develop a nomogram to predict reduced cardiac function for acute kidney injury (AKI) patients who received continuous renal replacement therapy (CRRT) after acute type A aortic dissection (ATAAD) surgery.

**Methods:**

This study was a retrospective analysis. ATAAD patients with preoperative normal ejection fraction (EF) and postoperative AKI with CRRT admitted between January 2014 and November 2021 were included. The reduced cardiac function was defined as EF <50%. The data were analyzed by the univariate and multivariate logistic regression analyses. A diagnostic model was established by a nomogram, and its discriminative performance was validated by the received operating characteristic (ROC) curve and concordance (C) statistic. The calibration of the diagnostic model was tested by calibration curves and the HosmerLemeshow test. The clinical utility was evaluated by the decision curve analysis (DCA).

**Result:**

In total, 208 patients were eligible for analysis, of which 98 patients with reduced cardiac function. The logistic regression analyses showed age ≥60 years old, history of coronary atherosclerotic disease, preoperative pericardial tamponade, and cardiopulmonary bypass time were risk factors for reduced cardiac function, which were further employed in the nomogram. As results, nomogram revealed a high predictive power (C statistic = 0.723, 0.654–0.792; the bootstrap-corrected concordance C statistic = 0.711, the area under the ROC curve = 0.723). The calibration curves showed good consistency between the predicted and the actual probabilities (calibration curve: Brier points = 0.208, Emax = 0.103, Eavg = 0.021; Hosmer-Lemeshow test, *P* = 0.476). DCA showed that the nomogram could augment net benefits and exhibited a wide range of threshold probabilities in the prediction of EF reduction.

**Conclusion:**

This nomogram is an effective diagnostic model for predicting the reduced cardiac function in postoperative ATAAD patients with AKI undergoing CRRT and can be used to protect postoperative renal functions and facilitate patient-specific care after ATAAD surgery.

## Introduction

Acute type A aortic dissection (ATAAD) is a catastrophic condition that need rapid diagnosis and treatment delivery (1, 2). Patients with ATAAD often suffer multiorgan complications after surgery due to severe systemic pathophysiological changes (3). Acute kidney injury (AKI) is one of the early postoperative complications, ranging from mild renal dysfunction to loss of kidney function requiring continuous renal replacement therapy (CRRT) (4). The incidence of AKI in postoperative ATAAD patients is from 20.2 to 44.9% (4–6). Studies have shown that the mortality of ATAAD patients with AKI is 15–30%, which is 10–20 times higher than that of those without AKI (6, 7). However, investigations that focus on the impact of postoperative AKI on the incidences of postoperative complications, including cardiovascular events such as reduced cardiac function, are still insufficient.

It is widely known that acute renal deterioration is mainly caused by water and sodium retention, increased volume load and other factors, resulting in cardiac dysfunction that is often associated with the increased central venous pressure (CVP) (8). The raised renal venous pressure induced by the elevated CVP, compression of renal tubules, the increased renal interstitial hydrostatic pressure, declined glomerular filtration rate (GFR), and water and sodium retention, thereby forming a vicious cycle (9–12). Importantly, cardiac and renal diseases interact in a complex bilateral and interdependent manner, especially in acute settings (13). Therefore, the cardiac function should be considered comprehensively when using CRRT for AKI after ATAAD.

In our clinical practice, when postoperative ATAAD patients with acute kidney injury are accompanied by reduced cardiac function, the vicious cycle of kidney and heart would lead to a worse prognosis. To date, accurate diagnosis of patients with CRRT and prediction of postoperative EF reduction after surgery for ATAAD have remained critical clinical challenges.

Hence, this study aimed to analyze the risk factors for the reduced cardiac function in postoperative ATAAD patients with AKI undergoing CRRT and establish a predictive model, the nomogram has been considered as an effective way to create a straightforward visual graph of a numerical predictive model that quantifies the risk of a clinical outcome. It can help clinicians make clinical decisions for targeted interventions and protect perioperative renal and cardiac functions after ATAAD surgery.

## Methods

### Patients and data collection

#### Study population

This study was a retrospective analysis. ATAAD patients from Beijing Anzhen Hospital with normal preoperative EF and postoperative CRRT for AKI, admitted between January 2014 and November 2021 were included in this study. Inclusion criteria were as follows: (1) patients' age ≥18 years old; (2) patients who underwent ATAAD surgery with moderate hypothermic circulatory arrest; (3) patients who underwent CRRT due to postoperative AKI; (4) preoperative echocardiography revealing normal EF (EF ≥50%); (5) patients who needed hospitalization in intensive care unit (ICU) for at least 3 days. Exclusion criteria were as follows: (1) abnormal EF revealed by preoperative echocardiography (EF <50%); (2) patients with a history of undergoing renal replacement therapy (RRT); (3) pregnant women; (4) moribund patients with expected death within 24 h; (5) patients with a history of chronic renal insufficiency (meeting any of the following criteria for more than 3 months: proteinuria ≥30 mg/24 h; albumin-to-creatinine ratio ≥3 mmol; abnormal urinalysis routine; electrolyte disorders caused by acute tubular necrosis; abnormal renal pathology; abnormal renal imaging; renal transplantation; estimated glomerular filtration rate [eGFR] <30 mL/min). This study was approved by the Institutional Ethics Committee of the Beijing Anzhen Hospital Affiliated to Capital Medical University.

AKI was diagnosed based on changes in the urine output, serum creatinine, or both, according to the Kidney Disease: Improving Global Outcomes (KDIGO) classification. Every patient had a urinary catheter to measure urine output every hour, and serum creatinine measurements were performed at least once daily. KDIGO classification stage 1 is defined by at least one of the following criteria: creatinine concentration of >0.3 mg/dl (26.5 μmol/liter) or >1.5–1.9 times the baseline creatinine level or urinary output <0.5 mL/kg/h for 6–12 h; KDIGO classification stage 2 is defined by at least one of the following criteria: 2-fold increase in serum creatinine from baseline (we used the serum creatinine at hospital admission as baseline serum creatinine) or urinary output <0.5 mL/kg/h for ≥12 h. KDIGO classification stage 3 is defined by at least one of the following criteria: (serum creatinine concentration of > 4 mg/dl (354 μmol/liter) or >3 times the baseline creatinine level; anuria for >12 h; oliguria for > 24 h).

#### Data collection

In the present study, surgical data were recorded. (1) Preoperative general data included age, gender, weight, height, time from the occurrence of dissection to the surgery, history of hypertension, type of dissection, the maximum diameter of the aorta, cardiac function grade, myocardial ischemia, aortic regurgitation, renal insufficiency, pleural effusion, pericardial tamponade, aortic rupture, shock, smoking history, diabetes, oral administration of β-receptor blockers, and calcium antagonists. (2) Intraoperative data included operation time, cardiopulmonary bypass (CPB) time, moderate hypothermic circulatory arrest time, minimum temperature, colloidal crystal input, and blood transfusion. (3) Postoperative data included blood pressure, CVP, mechanical ventilation time, ICU stay, blood transfusion volume, etc. Complications included pulmonary infections, respiratory failure, organ dysfunction, hemodynamic instability, arrhythmia, CRRT catheterization, anticoagulation-related bleeding, and acid-base imbalance. The urinary volume per hour, daily intake and output, blood creatinine, urea nitrogen, electrolyte, pH, and internal environment (whether acid-base balance) were postoperatively collected. Patients' treatment measures involved mechanical ventilation, vasoactive medications, fluid therapy, etc.

Grouping: All the included patients were divided into the reduced postoperative EF group (EF <50%) and normal postoperative EF group (EF ≥50%) according to whether the postoperative EF was reduced.

The timing of most patients undergoing CRRT initiated within 8 h of stage 3 AKI using the KDIGO classification or if any of the following absolute indications for RRT were present: serum urea level > 40 mmol/liter; Serum potassium concentration of > 6 mmol/liter despite medical treatment (bicarbonate and/or glucose-insulin infusion); pH < 7.15 in a context of pure metabolic acidosis (PaCO2 below 35 mmHg) or in a context of mixed acidosis with PaCO2 ≥ 50 mmHg without the possibility of increasing alveolar ventilation.

Proton pump inhibitors were routinely used after ATAAD surgery, and each patient was appointed to about 40 mg of esomeprazole magnesium enteric-coated per day. All patients used the above methods to prevent upper gastrointestinal bleeding. Diuretics are used in patients with oliguria, usually after 12 h of diuretics, there is no significant improvement in urine output and the elevated serum creatinine or urine output is at least at the KDIGO stage 2, CRRT is considered, patients are treated with standardized therapy and the use of these drugs has no significant difference between EF normal group and EF reduction group.

### Statistical analysis

Patients' baseline characteristics were expressed as frequency and percentage for categorical variables and as mean ± standard deviation or median and interquartile range (IQR) for continuous variables. Besides, the variance inflation factor (VIF) was used to evaluate all variables for collinearity.

**Figure 1:**
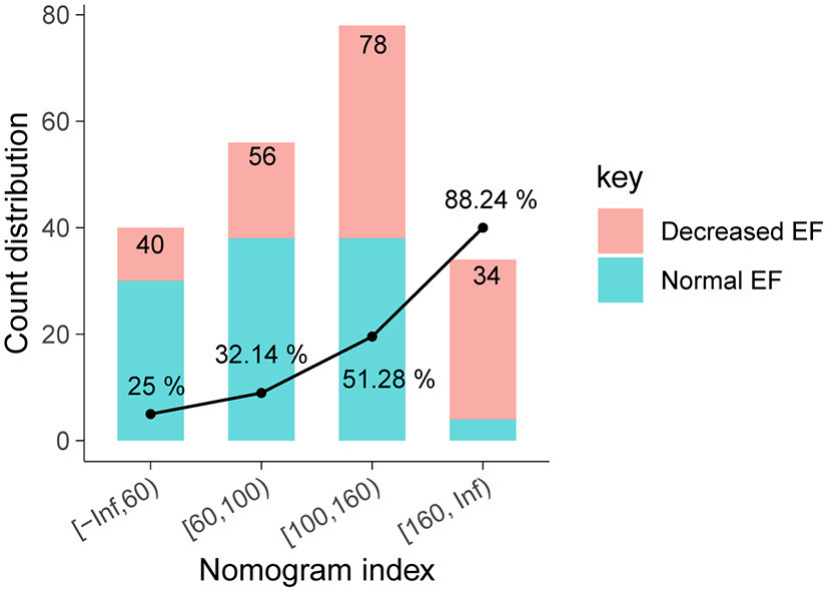
Supervised tree-like segmentation of EF.

The sample size calculation showed that an estimated 34 patients with a nomogram index higher than 160 would be needed to provide 95% power for detecting a minimum clinically meaningful Decreased EF rate of 88.24% with a two-side α of 0.05 when compared with patients with nomogram index lower than 160 (Figure 1).

As appropriate, binary data were examined using the Chi-squared test or the Fisher's exact test for significance. Event rates were calculated via the odds ratio (OR) and asymptotic 95% confidence intervals (CI). EF or lgEF is not normally distributed data. EF reduction or normal is the basis for this study's grouping, when comparing the continuous variables of two groups. Normally distributed data were compared for significance using the *t*-test. Abnormally distributed data were compared for significance using the Mann-Whitney *U* test, and median values were compared using the Hodges-Lehmann estimator of location shift with 95% CI. The significance of each variable was assessed by the univariate logistic regression analysis. Variables with *P* < 0.1 were imported into the multivariate logistic regression analysis to identify independent risk factors. Next, a nomogram was constructed to predict the probability of postoperative AKI in patients undergoing CRRT accompanied with the decreased EF using the “rms” package in R 4.1.2 software. The regression coefficients in the multivariate logistic regression analysis were proportionally converted to a point scale, and the total points were transformed into the predicted probabilities (14).

The performance of the nomogram was evaluated by discrimination and calibration. The discriminative ability of the model was reflected by the area under the receiver operating characteristic (ROC) curve, which is equal to the concordance statistic. Calibration was performed by a visual calibration plot, which was described through brier points, Emax, Eavg, etc. (15) An insignificant Hosmer-Lemeshow test also indicated a good calibration (*P* > 0.05). Decision curve analysis (DCA) was established to evaluate the clinical benefit of the nomogram by quantifying the net benefits along with the increase of threshold probabilities. The statistical analysis and graphics were performed with R 4.1.2 software. All tests were 2-tailed, and P <0.05 was considered statistically significant.

Indicators with missing values warranted interpolation by multiple imputations using the MICE package (16). We assumed that the data were missing at random (17); therefore, we performed predictive mean matching (18) to generate five complete imputed data sets that fit the logistic models. We have modified these parts in the Statistical Analysis already.

## Results

**Figure 2:**
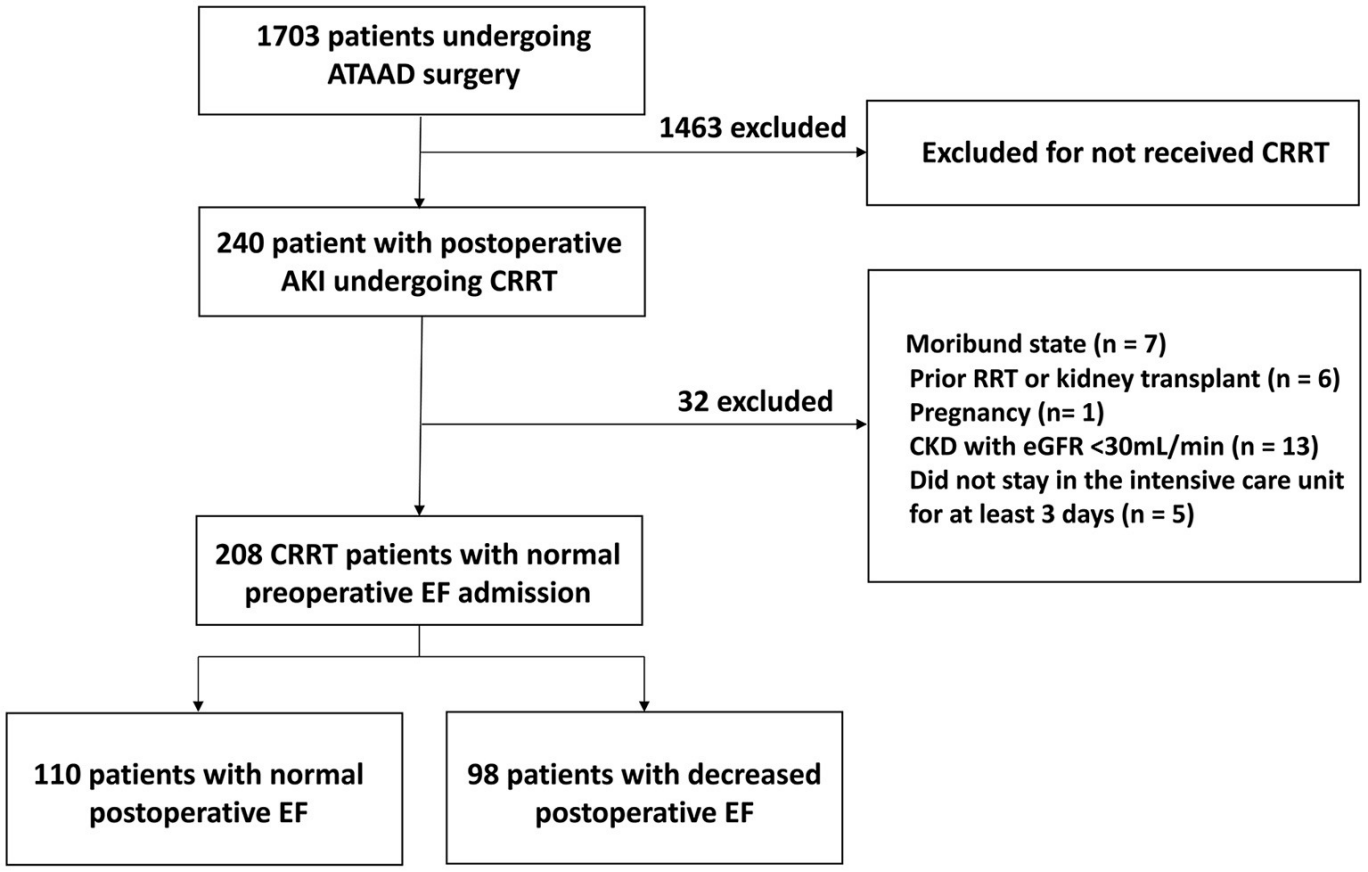
The study flowchart.

In total, 208 patients were eligible for analysis, of which 98 patients (47.1%) with reduced cardiac function (Figure 2). The mean EF in the normal EF group was 55.4 ± 4.9%, while 41.3 ± 5.2% in the reduced EF group (*P* < 0.001). The comparison of the baseline data showed that the mean age in the normal EF group was 51.6 ± 11.2, while 55.0 ± 10.7 in the reduced EF group (*P* = 0.02). In addition, the number of patients aged ≥60 years old in the reduced postoperative EF group was significantly higher than that in the normal postoperative EF group {age ≥60 years old: reduced postoperative EF group [40 (40.8%) cases] vs. normal postoperative EF group [24 (21.8%) cases], *P* = 0.003}. Significantly more patients had a history of coronary atherosclerotic disease (CAD) in the reduced postoperative EF group than that in the normal postoperative EF group {CAD history: reduced postoperative EF group [12 (12.2%) cases] vs. normal postoperative EF group [4 (3.3%) cases], *P* = 0.004}. Significantly more patients had a history of diabetes in the reduced postoperative EF group than that in the normal postoperative EF group {diabetes history: reduced postoperative EF group [16 (16.3%) cases] vs. normal postoperative EF group [10 (8.2%) cases], *P* = 0.04}. Significantly more patients had preoperative pericardial tamponade in the reduced postoperative EF group than that in the normal postoperative EF group {preoperative pericardial tamponade: reduced postoperative EF group [26 (26.5%) cases] vs. normal postoperative EF group [8 (7.3%) cases], *P* < 0.001}. Therefore, age, history of CAD, diabetes, and preoperative pericardial tamponade were involved in the multivariate logistic regression analyses. There were no significant differences in other baseline data between the two groups (Table 1).

**Table 1:** Comparison of the baseline characteristics between the normal postoperative EF group and the reduced postoperative EF group.

**Variables**	**Normal postoperative EF group**	**Reduced postoperative EF group**	***P***
Sex (male/female)	74/36	70/28	0.82
Age (years, x ± S)	51.6 ± 11.2	55.0 ± 10.7	***0.02***
Age <40 years old (%)	16 (14.5)	6 (6.1)	
Age between 40–59 years old (%)	70 (63.6)	52 (53.1)	
Age ≥60 years old (%)	24 (21.8)	40 (40.8)	
BMI (kg/m^2^)	25.3 ± 4.2	25.4 ± 3.9	0.91
Hypertension (%)	102 (83.6)	74 (75.5)	0.15
CAD (%)	4 (3.3)	12 (12.2)	***0.004***
Diabetes (%)	10 (8.2)	16 (16.3)	***0.04***
Preoperative EF (%)	63.9 ± 6.1	61.0 ± 6.9	0.48
Worst postoperative EF (%)	55.4 ± 4.9	41.3 ± 5.2	***<0.001***
Preoperative pericardial tamponade (%)	8 (7.3)	26 (26.5)	***<0.001***
Dissection involving the coronary opening (%)	24 (21.8)	16 (16.3)	0.32
Creatinine (μmol/L, x ± S)	111.0 ± 44.1	105.5 ± 45.9	0.45
Urea nitrogen (mmol/L, x ± S)	8.8 ± 4.1	7.7 ± 2.2	0.07
Albumin (g/L, x ± S)	38.9 ± 7.5	38.3 ± 7.0	0.56
Myohemoglobin (μg/L, x ± S)	369.4 ± 97.6	203.2 ± 72.5	0.23
Leukocytes (10^9^/L, x ± S)	13.2 ± 3.7	12.8 ± 4.7	0.83
Hemoglobin (g/L, x ± S)	135.7 ± 15.2	132.1 ± 20.0	0.11
ICU stay (days, x ± S)	8.4 ± 4.0	9.4 ± 5.9	0.11
Length of hospital stay (days, x ± S)	17.4 ± 10.9	17.1 ± 10.7	0.84

*BMI, body mass index; CAD, coronary atherosclerotic disease; ICU, intensive care unit. The bold values indicate the values of p < 0.05*.

Comparison of intraoperative data between the two groups revealed that the CPB time in the reduced postoperative EF group was longer than that in the normal postoperative EF group [CPB time: reduced postoperative EF group (233.5 ± 56.3 min) vs. normal postoperative EF group (211.5 ± 53.7 min), *P* = 0.004]. Furthermore, significantly more patients with CPB time greater than 240 min in the reduced postoperative EF group than that in the normal postoperative EF group {CPB >240 min: reduced postoperative EF group [38 (38.8%) cases] vs. normal postoperative EF group [28 (25.5%) cases], *P* = 0.04}. Comparison of other intraoperative data, including the type of surgery, operation time, aorta occlusion time, moderate hypothermic circulatory arrest time, and intraoperative blood transfusion volume, showed no significant difference (Table 2). Therefore, CPB time was included in the multivariate logistic regression analysis.

**Table 2:** Comparison of the intraoperative variables between the normal postoperative EF group and the reduced postoperative EF group.

**Variables**	**Normal postoperative EF group**	**Reduced postoperative EF group**	***P***
Type of surgery			
Bentall procedure (%)	48 (43.6)	48 (48.9)	0.44
Total aortic arch replacement (%)	106 (96.4)	92 (93.9)	0.40
Partial aortic arch replacement (%)	2 (1.8)	6 (6.1)	0.11
Combined CABG (%)	8 (7.3)	6 (6.1)	0.74
Operative time (min, x ± S)	410.4 ± 207.7	429.9 ± 207.8	0.49
CPB time (min, x ± S)	211.5 ± 53.7	233.5 ± 56.3	***0.004***
CPB time <180 min (%)	28 (25.5)	16 (16.3)	
CPB time between 180–240 min (%)	54 (49.1)	44 (44.9)	
CPB time >240 min (%)	28 (25.5)	38 (38.8)	
Aortic cross-clamp time (min, x ± S)	124.8 ± 36.7	122.8 ± 35.8	0.69
MHCA time (min, x ± S)	21.7 ± 7.2	23.1 ± 9.0	0.22
Intraoperative infusion of RBC (u, Q1, Q3)	4.0 (1.5, 6.5)	4.0 (0.0, 6.5)	0.34
Intraoperative infusion of platelet (u, Q1, Q3)	0 (0, 0)	0 (0, 0)	0.64
Intraoperative infusion of plasma (u, Q1, Q3)	400.0 (0, 600.0)	400.0 (0, 400.0)	0.77

*CABG, coronary artery bypass grafting; CPB, cardiopulmonary bypass; MHCA, moderate hypothermic circulatory arrest; RBC, red blood cells. The bold values indicate the values of p < 0.05*.

Comparison of the laboratory indicators between the reduced postoperative EF group and the normal postoperative EF group at the beginning of CRRT showed no significant difference (Table 3).

**Table 3:** Comparison of laboratory indicators upon initiation of CRRT between the normal postoperative EF group and the reduced postoperative EF group.

**Variables**	**Normal postoperative EF group**	**Reduced postoperative** **EF group**	**P**
Albumin (g/L, x ± S)	30.7 ± 10.4	29.7 ± 7.2	0.69
Leukocytes (g/L, x ± S)	17.5 ± 7.7	17.4 ± 7.6	0.35
BUN (ummol/L, x ± S)	20.5 ± 11.6	20.6 ± 11.9	0.53
Creatinine (mmol/L, x ± S)	298.9 ± 160.2	262.7 ± 147.2	0.21
Hemoglobin (g/L, x ± S)	92.2 ± 17.8	92.9 ± 18.1	0.32
Lactic acid (mmol/L, x ± S)	5.0 ± 4.2	4.7 ± 3.7	0.30
Serum potassium (mmol/L, x ± S)	5.4 ± 2.9	6.4 ± 2.8	0.28
Bicarbonate (mmol/L, x ± S)	24.9 ± 3.8	24.5 ± 3.9	0.65

*BUN, blood urea nitrogen. The bold values indicate the values of p < 0.05*.

Comparison of postoperative complications and transfusion data during ICU stay between the two groups: The proportion of liver dysfunction in the reduced postoperative EF group was significantly higher than that in the normal postoperative EF group {liver dysfunction: reduced postoperative EF group [22 (22.4%) cases] vs. normal postoperative EF group [13 (11.8%) cases], *P* = 0.04}. Liver dysfunction is mainly closely correlated with right heart failure, and right heart failure can also affect left heart function, leading to reduced EF; in addition, when left heart failure also leads to the right heart failure, liver dysfunction may occur. Therefore, it is difficult to strictly define the association of liver dysfunction with EF reduction. Thus, liver dysfunction was not involved in the multivariate logistic regression analysis. There were no significant differences in other complications and the volume of blood transfusion during ICU stay between the two groups (Table 4).

**Table 4:** Comparison of postoperative complications and transfusion data during ICU between the normal postoperative EF group and the reduced postoperative EF group.

**Variables**	**Normal postoperative EF group**	**Reduced postoperative** **EF group**	***P***
Liver dysfunction (%)	13 (11.8)	22 (22.4)	***0.04***
Paraplegia inferior (%)	16 (13.1)	6 (6.1)	0.09
Catheter-related bloodstream infection (%)	4 (3.6)	4 (4.1)	0.87
Lung infection (%)	22 (20.0)	20 (20.4)	0.94
Gastrointestinal bleeding (%)	12 (10.9)	6 (6.1)	0.22
Infusion of RBC (u, Q1, Q3)	12.0 (6.0, 18.0)	14.0 (5.5, 22.5)	0.52
Infusion of plasma (u, Q1, Q3)	400.0 (0,600.0)	400.0 (0,600.0)	0.99
Infusion of platelet (u, Q1, Q3)	3.0 (1.0, 5.0)	2.5 (0.0, 4.0)	0.44

*Liver dysfunction, alanine aminotransferase or aspartate aminotransferase more than 10 times the upper limit of normal value; RBC, red blood cells. The bold values indicate the values of p < 0.05*.

### Selected factors for model

The variables used in the analysis were important clinical characteristics and risk factors. The results of the univariate analysis of variables with *P* < 0.1 were imported into the multivariate logistic regression analysis. The present study showed that the variables, such as age, history of CAD, history of diabetes, CPB time, and preoperative pericardial tamponade had a significant difference in the univariate logistic regression analysis (*P* < 0.1, Table 5) and were therefore imported into the multivariate logistic regression analysis. The results showed that age (*P* = 0.02), history of CAD (OR, 5.26; 95% CI, 1.61–17.25; *P* = 0.006), CPB time (*P* = 0.04), and preoperative pericardial tamponade (OR, 4.55; 95% CI, 1.86–11.13; *P* = 0.001) were independent risk factors associated with reduced postoperative EF in patients with AKI undergoing CRRT after ATAAD (Table 6).

**Table 5:** The results of univariate logistic regression analysis showing the risk factors of EF reduction in patients with AKI undergoing CRRT after ATAAD surgery.

**Variables**	***P***	**OR (95%CI)**	**AUC**
**Age**	***0.006***		0.60
Age <40 years old (reference group)			
Age between 40–59 years old	0.18	1.98 (0.73–5.41)	
Age ≥ 60 years old	***0.006***	4.44 (1.53–12.91)	
History of CAD	***0.004***	5.17 (1.67–16.05)	0.56
History of diabetes	***0.04***	2.49 (1.01–6.10)	0.55
Preoperative pericardial tamponade	***<0.001***	4.60 (1.97–10.75)	0.60
**CPB time**	***0.04***		0.62
CPB time <180 min (reference group)			
CPB time between 180–240 min	0.34	1.43 (0.69–2.96)	
CPB time >240 min	***0.03***	2.38 (1.08–5.20)	

*CAD, coronary atherosclerotic disease; CPB, cardiopulmonary bypass; OR, odds ratio; AUC, area under curve. The bold values indicate the values of p < 0.05*.

**Table 6:** The results of multivariate logistic regression analysis showing the independent risk factors of EF reduction in patients with AKI undergoing CRRT after ATAAD surgery.

**Variables**	***P***	**OR (95%*CI)***
**Age**	***0.02***	
Age <40 years old (reference group)		
Age between 40–59 years old	0.08	2.84 (0.90–8.94)
Age ≥ 60 years old	***0.006***	5.55 (1.63–18.94)
History of CAD	***0.006***	5.26 (1.61–17.25)
Preoperative pericardial tamponade	***0.001***	4.55 (1.86–11.13)
**CPB time**	***0.04***	
CPB time <180min (reference group)		
CPB time between 180–240 min	0.74	1.14 (0.51–2.55)
CPB time > 240 min	***0.04***	2.56 (1.07–6.13)

*CAD, coronary atherosclerotic disease; CPB, cardiopulmonary bypass; OR, odds ratio. The bold values indicate the values of p < 0.05*.

### Predictive performance of the nomogram

**Figure 3:**
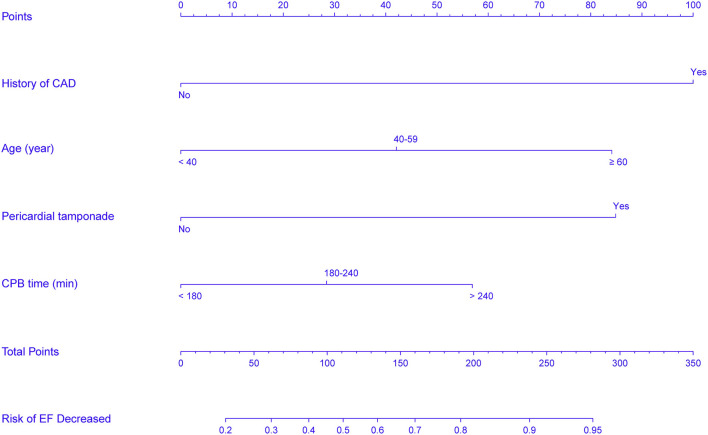
Nomogram predicts EF reduction risk in patients with AKI undergoing CRRT after ATAAD surgery. The nomogram was established to predict the risk of decreased EF in patients with CRRT based on 4 independent prognostic factors. The value of each of variable wasgiven a score on the point scale axis. The total score can be calculated by summation of single scores. We can estimate the probability of decreased EF by projecting the total score to the lower total point scale.

**Figure 4:**
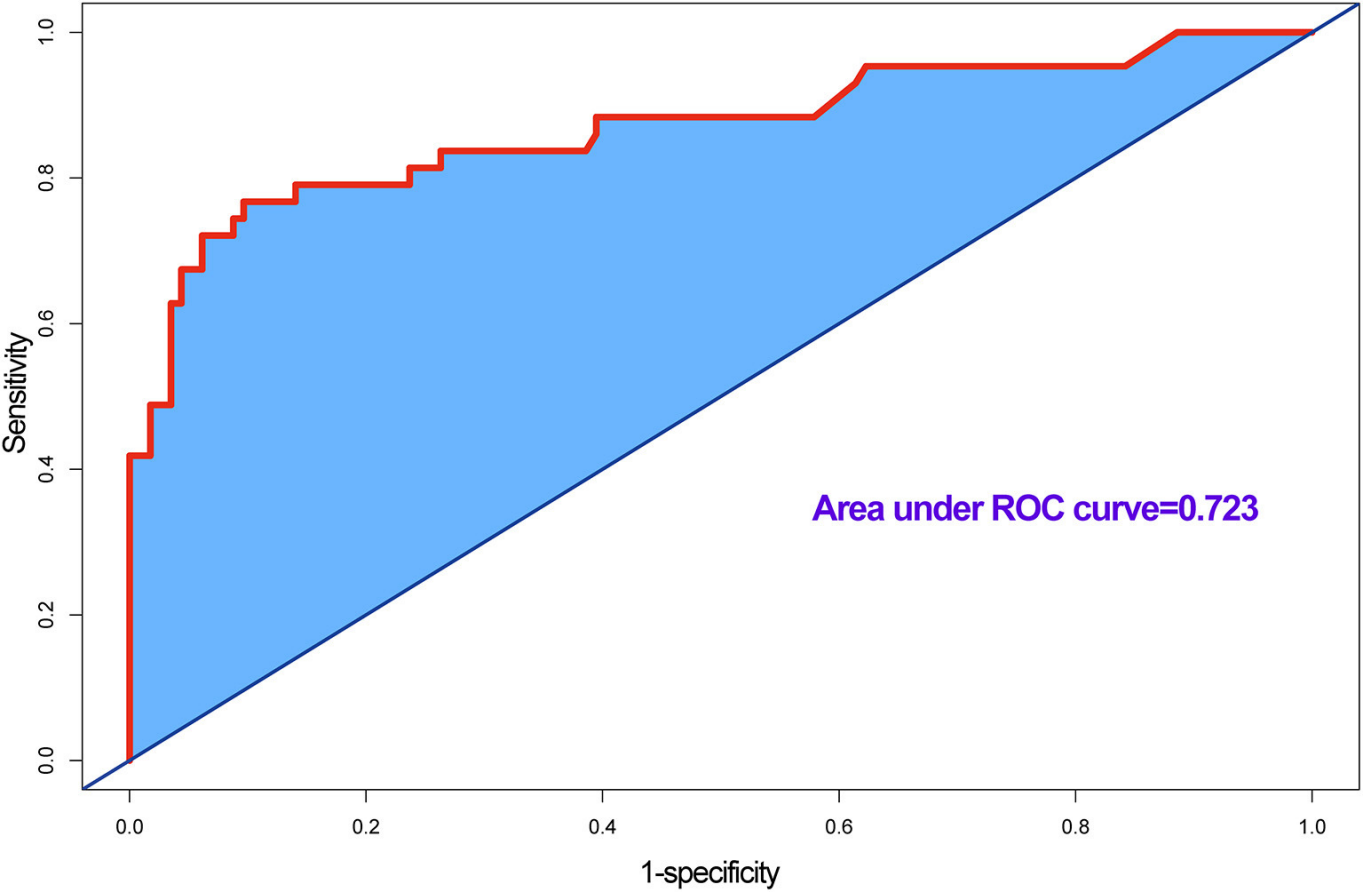
Receiver operating characteristic (ROC) curve for evaluating the discrimination performance of the model, area under ROC curve was 0.723, and concordance (C) statistic was 0.723.

**Figure 5:**
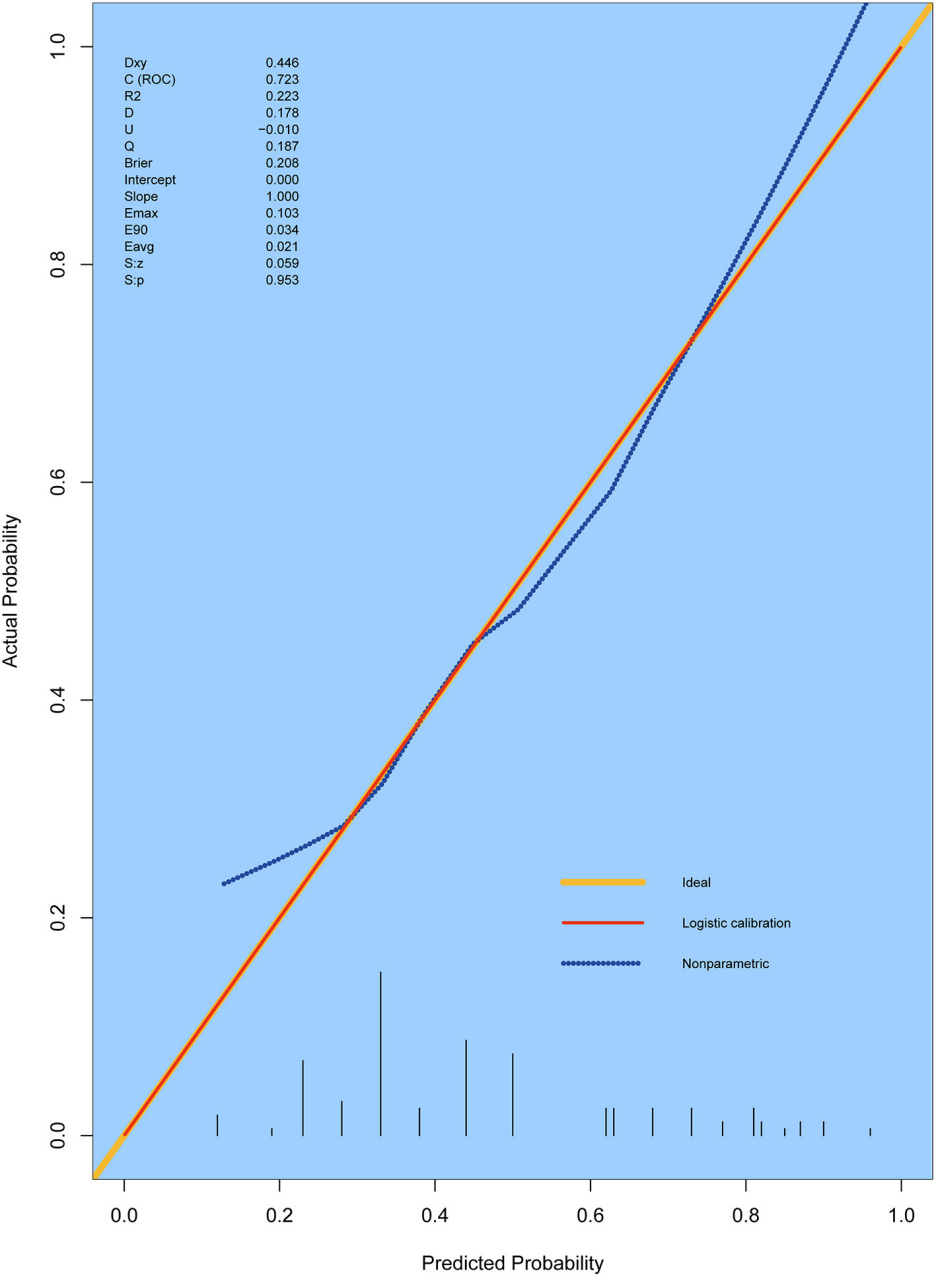
Calibration curves for the prediction model. The curves describe the calibration of the nomogram in terms of the agreement between predicted risks (X-axes) and actual outcomes (Y-axis). The diagonal line indicates a perfect prediction by an ideal model. E max, maximum error; E aver, average error.

In accordance with the results of the multivariate logistic regression analysis, a nomogram was established to predict postoperative AKI in patients undergoing CRRT accompanied with reduced EF after ATAAD surgery, including 4 significant independent risk factors (age, history of CAD, preoperative pericardial tamponade, and CPB time) (Figure 3). The total score was calculated by summation of the single scores, which was used to estimate the probability of reduction of EF. The discrimination of the predictive model was assessed using concordance statistic of 0.723 (95% CI, 0.654–0.792) and a bootstrap-corrected concordance statistic of 0.711, in which the area under ROC value was also 0.723 (Figure 4). The calibration curve showed that the predicted probabilities of decreased EF well matched with the actual prevalence rate [calibration curve: Brier points = 0.208, Emax (maximal error) = 0.103, Eavg (average error) = 0.021] (Figure 5), and the Hosmer-Lemeshow test (*P* = 0.476) also demonstrated good calibration.

**Figure 6:**
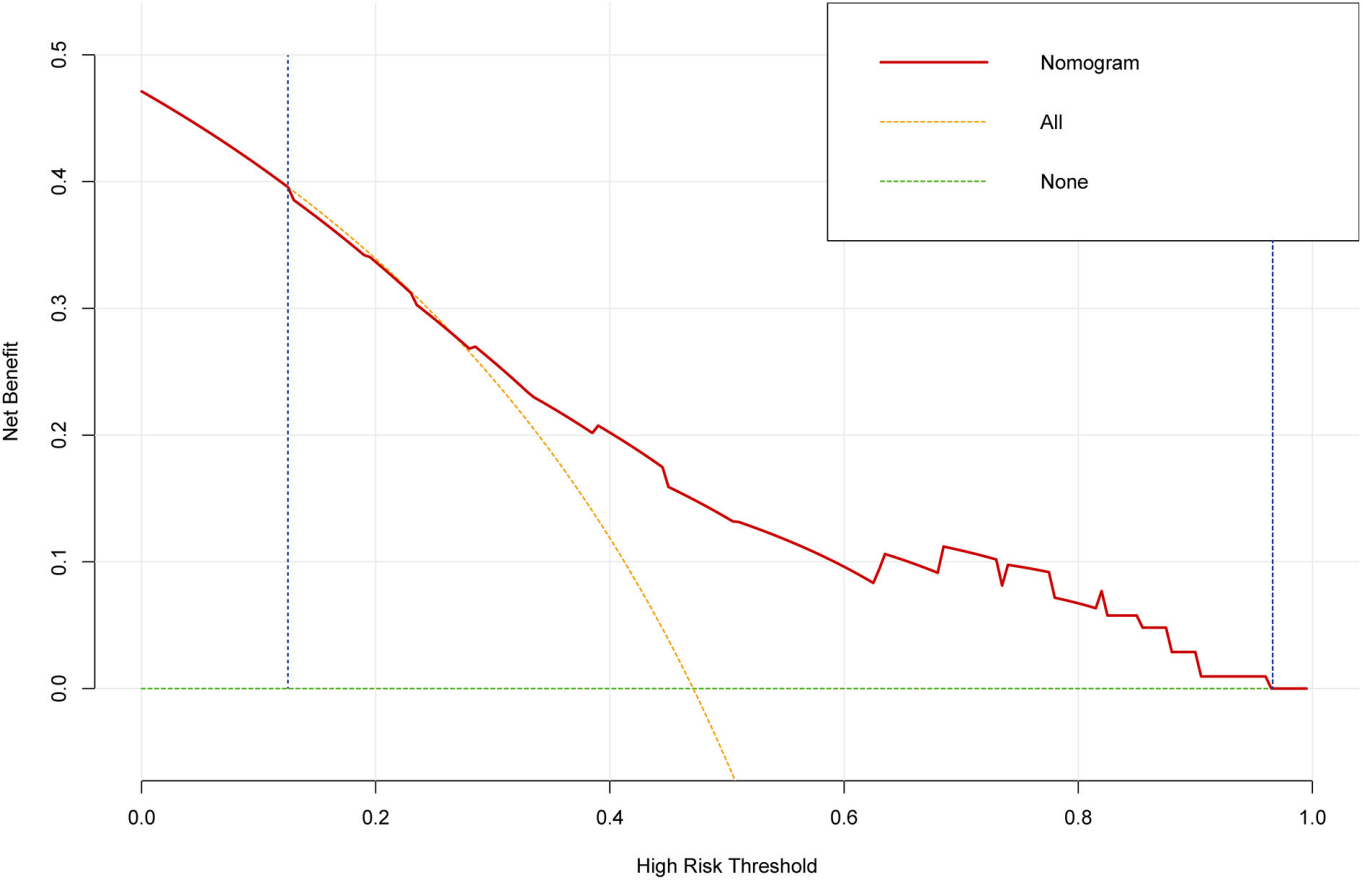
The decision curve analysis (DCA) for the prediction model. The DCA showed that the nomogram could augment net benefits and exhibited a wide range of threshold probabilities in the EF reduction.

DCA is usually used to assess the clinical net benefit of the prediction model. In this study, DCA revealed that the nomogram could augment net benefts and exhibited a wide range of threshold probabilities in EF reduction (Figure 6). DCA also showed that the nomogram had a good clinical utility.

## Discussion

In the present study, the important contributors to the risk of decreased EF were the history of CAD and preoperative pericardial tamponade. AKI is a common complication after ATAAD surgery. The mortality of patients with AKI undergoing CRRT after ATAAD surgery was significantly higher than that of patients without AKI. Acute deterioration of renal function mainly results in cardiac dysfunction. The mechanisms leading to cardiac dysfunction can be summarized as follows (19–21): (1) When AKI occurs, GFR decreases with water and sodium retention, volume load increases, and blood pressure elevates, resulting in acute left heart failure or acute pulmonary edema. (2) Acidosis and uremia can reduce myocardial contractility. Hyperkalemia can cause arrhythmia, and cardiac arrest and uremia can lead to pericarditis. (3) Ischemic kidney injury can activate inflammatory pathways, aggravate myocardial apoptosis, and induce heart failure that typically coexists with the increased CVP. The elevated renal venous pressure caused by the increased CVP can lead to the raised renal interstitial pressure, compressing renal tubules, resulting in the increased renal interstitial hydrostatic pressure, declined GFR, and water and sodium retention, thereby forming a vicious cycle. Thus, cardiac failure and renal dysfunction can coexist and affect each other, seriously influencing patients' prognoses. No preoperative reduced EF was found in the majority of ATAAD patients. When renal failure requires CRRT with postoperative EF reduction, it is mainly associated with patients' clinical severe conditions and poor prognosis, indicating the necessity of special attention. Therefore, identifying the risk factors in these patients is vital to improving their prognosis, reducing mortality rates, and improving their quality of life.

Several factors have been reported to be associated with high mortality during CRRT or after ATAAD surgery (9, 22). However, few studies have described risk factors for postoperative EF reduction in patients with AKI undergoing CRRT after ATAAD surgery. Therefore, it is essential to establish a reliable model for predicting the prognosis of these patients, playing an essential role in the monitoring and management of patients.

Therefore, the present research was of great clinical application value because the model could predict postoperative EF reduction in patients with AKI undergoing CRRT after ATAAD surgery. Among the available prediction tools, the nomogram can easily quantify the risk of decreased EF and have promising discrimination and calibration in predicting outcomes. It is noteworthy that the current study was the first to construct a quantitative nomogram to predict the probability of postoperative EF reduction in patients with AKI undergoing CRRT after ATAAD surgery. In the established nomogram, the history of CAD was the most significant contributor to the risk of postoperative EF reduction, followed by the preoperative pericardial tamponade and age, and CPB time showed the minimum effect on the probability of postoperative EF reduction.

The results of the present study suggested that age was a predictive factor for the postoperative EF reduction in AKI patients undergoing CRRT. The immune function in elderly patients is declined with degenerative physiological functions. Moreover, most of them are accompanied by various metabolic diseases, such as hypertension, hyperlipidemia, and diabetes. If the preoperative EF is normal, it is easier to cause a reduction of EF by surgery and other initiative factors. Studies have shown that the mortality of AKI patients aging over 65 years old with a need for CRRT in the ICU was more than 70%, that of patients aging over 80 years old was as high as 76%, and the death risk was significantly higher than that of patients aging under 50 years old. The prognosis of patients undergoing CRRT was worse with the increase of age. The results mentioned above (23, 24) also supported the findings of the present study. The present study showed that the risk of postoperative reduced EF in patients undergoing CRRT increased with age, and the prognosis of patients aged ≥60 years old was mainly more worrisome.

The present study also revealed that the history of CAD was a predictive factor for postoperative EF reduction in patients undergoing CRRT. The pathogenesis of ATAAD complicated with CAD could be based on the following explanations: (1) the two diseases have common risk factors, including hypertension, dyslipidemia, and negative living habits, such as smoking and drinking. These risk factors alone or synergistically promote atherosclerosis in blood vessels, including the aorta (2). (2) Inflammatory response plays an important role in the pathophysiological mechanism of atherosclerosis in the aorta and coronary artery (25). Studies have shown that even after surgical treatment, the nosocomial mortality of patients with ATAAD and coronary hypoperfusion or severe coronary artery disease was still higher than 50% (26, 27). When ATAAD patients have a history of CHD, cardiac function may deteriorate by the surgical strike even if the preoperative EF is normal. As mentioned above, cardiac and renal functions can affect each other and form a vicious cycle. Patients with AKI undergoing CRRT after ATAAD surgery have poor renal function. The possibility of postoperative EF reduction is higher, leading to more complicated therapies and causing additional clinical challenges.

It was also found that preoperative pericardial tamponade was a predictive factor for postoperative EF reduction in patients undergoing CRRT. Preoperative pericardial tamponade can significantly reduce cardiac output, affect hemodynamic stability, and threaten patients' life in serious clinical conditions. ATAAD patients with preoperative pericardial tamponade need to receive surgical treatment as soon as possible (28, 29). These patients are accompanied by a high possibility of postoperative EF reduction. Therefore, it is essential to pay further attention to their circulation function and closely monitor their vital signs to prevent the deterioration of the conditions. Identifying the above-mentioned predictive factors is beneficial for improving the success rate of treatment of patients undergoing CRRT with reduced EF after ATAAD surgery and timely adjusting the treatment plan to improve the prognosis.

It was also found that the CPB time was a predictor of postoperative EF reduction in the mentioned patients. Aortic surgery is difficult, and the surgical technique is complex, and prolonged CPB time may not ensure normal renal blood flow and perfusion pressure. The CPB may change renal perfusion from pulsatile to non-pulsatile and contribute to ischemic and hypoxic renal injuries (26). It may also cause the release of catecholamines, resulting in strong renal vasoconstriction and reduced renal perfusion, causing AKI. Furthermore, significant contacts between blood and artificial materials, and inflammatory cells, such as neutrophils are activated, causing the release of various inflammatory mediators, chemokines, and proteases, as well as the generation of oxygen free radicals, resulting in a systemic inflammatory response (30). Therefore, after the extension of CPB time, patients are prone to cardiac dysfunction. Moreover, all surgeries for ATAAD were performed under moderate hypothermic circulatory arrest in the present study, with more pronounced ischemia of the organ, in which microcirculatory embolization and altered hemodynamics were the leading causes of end-organ damage.

The recent realization that various classes of approved anti-hyperglycemic agents may have divergent effects on cardiac function, and that some classes of agents might actually reduce heart failure risk, has led to a closer examination of the relationship between treatments and outcomes (31). For example, treatment with GLP-1 receptor agonists has beneficial effects on cardiovascular, mortality, and kidney outcomes in patients with type 2 diabetes (32). We measured blood glucose every 4 h in the ICU. We maintained the patient's blood glucose at around 180 mg/dl by pumping insulin or increasing sugar-containing infusions. Furthermore, hypotensive drugs are also influential factors for patients' cardiac function, Some researchers found that lower systolic blood pressure was associated with worse outcomes in patients with heart failure and reduced EF (33, 34). We did not calculate the specific dose of the applied hypotensive drugs and tried to keep the patient's circulation as stable as possible by adjusting the drugs and maintaining the mean arterial pressure at 65–90 mmHg. Due to the specificity management of the center for cardiac intensive care, it is difficult to collect them specifically, and we will continue to explore them in our future studies.

The present study has some limitations. First, although internal validation of the model resulted in optimal discrimination and good calibration, the generalizability of the nomogram still requires external validation, particularly from other countries, considering the differences in clinical behaviors and epidemiology that exist among different ethnic groups. Second, the prediction model was established retrospectively, and further prospective study is required to verify the reliability of the established model. Third, the accuracy of the nomogram did not reach a noticeable level of reliability. In the case of making critical clinical decisions, the misdiagnosis rate is still noteworthy.

## Conclusions

This nomogram is an effective diagnostic model for predicting the reduced cardiac function in postoperative ATAAD patients with AKI undergoing CRRT and can be used to protect postoperative renal functions and facilitate patient-specific care after ATAAD surgery.

## Data availability statement

The original contributions presented in the study are included in the article/supplementary material, further inquiries can be directed to the corresponding author/s.

## Ethics statement

This study was approved by the Institutional Ethics Committee of the Beijing Anzhen Hospital (No. KS2019034-3). The patients/participants provided their written informed consent to participate in this study.

## Author contributions

RJ carried out the studies, participated in collecting data, and drafted the manuscript. XL and ML participated in the acquisition, analysis, or interpretation of data. NL, LS, and JZ reviewed and edited it. All authors contributed to the interpretation of the data, the completion of figures and tables, and read and approved the final manuscript.

## Funding

The study was supported by the Beijing Municipal Science & Technology Commission (No. Z191100006619095).

## Conflict of interest

The authors declare that the research was conducted in the absence of any commercial or financial relationships that could be construed as a potential conflict of interest.

## Publisher's note

All claims expressed in this article are solely those of the authors and do not necessarily represent those of their affiliated organizations, or those of the publisher, the editors and the reviewers. Any product that may be evaluated in this article, or claim that may be made by its manufacturer, is not guaranteed or endorsed by the publisher.

## Abbreviations

BMI: Body Mass Index
ATAAD: Acute Type A Aortic Dissection
AKI: Acute Kidney Injury
CRRT: Continuous Renal Replacement Therapy
CABG: Coronary Artery Bypass Grafting
CAD: Coronary Atherosclerotic Disease
CPB: Cardiopulmonary Bypass
MHCA: Moderate and Hypothermia Circulation Arrest
EF: Ejection Fraction
ILI: Ischemic Liver Injury
BUN: Blood Urea Nitrogen
RBC: Red Blood Cell
ICU: Intensive Care Unit.
